# Atorvastatin in the treatment of Lithium-induced nephrogenic diabetes insipidus: the protocol of a randomized controlled trial

**DOI:** 10.1186/s12888-018-1793-9

**Published:** 2018-07-16

**Authors:** Jocelyn Fotso Soh, Susana G. Torres-Platas, Serge Beaulieu, Outi Mantere, Robert Platt, Istvan Mucsi, Sybille Saury, Suzane Renaud, Andrea Levinson, Ana C. Andreazza, Benoit H. Mulsant, Daniel Müller, Ayal Schaffer, Annemiek Dols, Pablo Cervantes, Nancy CP Low, Nathan Herrmann, Birgitte M. Christensen, Francesco Trepiccione, Tarek Rajji, Soham Rej

**Affiliations:** 1 Geri-PARTy Research Group, Jewish General Hospital/Lady Davis Institute, McGill University, 4333 Cote Ste-Catherine, Montreal, QC, H3T, 1E4, Montreal, Canada; 2 Douglas Mental Health University Institute and Department of Psychiatry, McGill University, Montreal, Canada; 3 Department of Epidemiology, Biostatistics and Occupational Health, McGill University Health Centre, Montreal, Canada; 4 Division of Nephrology, University Health Network, University of Toronto (UofT), Toronto, Canada; 5 Department of Psychiatry, Centre for Addiction and Mental Health & Department of Psychiatry, University of Toronto, Toronto, Canada; 6 Department of Psychiatry, Sunnybrook Research Institute, University of Toronto, Toronto, Canada; 7 Department of Psychiatry, GGZ, Geest, Amsterdam, the Netherlands; 8 Department of Psychiatry, McGill University Health Centre, Montreal, Canada; 9 Department of Biomedicine, University of Aarhus, Aarhus, Denmark; 10 Division of Nephrology, University of Naples, Naples, Italy

**Keywords:** Lithium, Nephrogenic diabetes insipidus, Kidney function, Atorvastatin, Placebo, Urinary osmolality, Randomized clinical trial

## Abstract

**Background:**

Lithium is the gold-standard treatment for bipolar disorder, is highly effective in treating major depressive disorder, and has anti-suicidal properties. However, clinicians are increasingly avoiding lithium largely due to fears of renal toxicity. Nephrogenic Diabetes Insipidus (NDI) occurs in 15–20% of lithium users and predicts a 2–3 times increased risk of chronic kidney disease (CKD). We recently found that use of statins is associated with lower NDI risk in a cross-sectional study. In this current paper, we describe the methodology of a randomized controlled trial (RCT) to treat lithium-induced NDI using atorvastatin.

**Methods:**

We will conduct a 12-week, double-blind placebo-controlled RCT of atorvastatin for lithium-induced NDI at McGill University, Montreal, Canada. We will recruit 60 current lithium users, aged 18–85, who have indicators of NDI, which we defined as urine osmolality (UOsm) < 600 mOsm/kg after 10-h fluid restriction. We will randomize patients to atorvastatin (20 mg/day) or placebo for 12 weeks. We will examine whether this improves measures of NDI: UOsm and aquaporin (AQP2) excretion at 12-week follow-up, adjusted for baseline.

**Results:**

Not applicable.

**Conclusion:**

The aim of this clinical trial is to provide preliminary data about the efficacy of atorvastatin in treating NDI. If successful, lithium could theoretically be used more safely in patients with a reduced subsequent risk of CKD, hypernatremia, and acute kidney injury (AKI). If future definitive trials confirm this, this could potentially allow more patients to benefit from lithium, while minimizing renal risk.

**Trial registration:**

ClinicalTrials.gov NCT02967653. Registered in February 2017.

## Background

Lithium remains the gold standard treatment for bipolar disorder and is associated with a better treatment response in 30–40% of patients compared to other bipolar pharmacotherapies [1, 2]. Lithium is also effective in treatment-resistant depression [1], has been associated with reduced suicidality [3], and is even being investigated in a number of neurological conditions such as dementia and stroke [4, 5]. Lithium is valuable considering the difficulty in achieving and maintaining symptomatic remission in the majority of patients with mood disorders [6]. Furthermore, lithium responders often do not respond well to alternative pharmacotherapies [5]. Rates of disability in people with mood disorders contribute to significant personal, family and societal costs: estimates of these total costs in the U.S. range from $US30–40 billion [7, 8].

Despite the effectiveness of lithium, many clinicians avoid using it, with prescribing rates declining markedly in the past 2 decades, particularly in America where only 8% of bipolar disorder patients receive lithium [9]. This is likely in large part due to the perception of serious potential adverse effects associated with lithium use compared to other treatment options [10].

Of particular clinical concern has been lithium’s potentially irreversible effects on kidney function: in addition to acute kidney injury (AKI), lithium has also been associated with a 2 times increased risk of chronic kidney disease (CKD) [11–13]. CKD, in turn, is associated with increased mortality, physical health co-morbities, decreased quality of life, family/caregiver burden, and major health care costs [14].

The exact mechanism whereby lithium causes CKD is not known. The most prominent pathology seen in patients with CKD attributed to lithium is interstitial nephropathy [15]. This points to a potential link between lithium related NDI and CKD. NDI is characterized by excessive production of dilute urine (> 3 L/24 h) due to tubular resistance to antidiuretic hormone [16]. Half of lithium users have difficulty concentrating urine: e.g. urine osmolality (UOsm) < 600 mOsm/kg following overnight water restriction [15]. Lithium-induced NDI is problematic: in addition to being related to frequent AKI and hypernatremia [17], NDI is also associated with a 2–3 times increased risk of chronic kidney disease (CKD) [12, 17]. Thus, treating lithium-induced NDI could potentially help prevent CKD.

Presently, amiloride is the only medication with evidence from small randomized controlled trials (RCTs) (*n* = 9, *n* = 11) to treat NDI, with a handful of other diuretics and non-steroidal anti-inflammatory agents (NSAIDs) having even lower-quality evidence [15, 18]. However, these drugs have been associated with acute lithium-level elevations of up to 33–50%, which could increase the risk of acute central nervous system and renal toxicity [19, 20]. Therefore, there is a need for novel, well-tolerated treatments for lithium-induced NDI.

In a recent cross-sectional study, we found that statins were associated with a lower risk of NDI amongst lithium users (*n* = 71). In this study, lithium users who were also using statins were at lower risk (0%; 0/17) of developing NDI compared to lithium users who were not on statins (20.4%; 11/54) [21]. Two mice studies also demonstrated the effectiveness of statins in genetic forms of NDI, where statins increased the expression of membrane aquaporin 2 in renal tubules and doubled the urine osmolality [22, 23]. Atorvastatin and other statins are well-tolerated and have minimal adverse effects on cognition and mood [24], but have positive effects on cardiovascular risk prevention [9], making it a palatable medication intervention for patients with mental disorders using lithium. In this present paper, we describe the first RCT to evaluate the effectiveness of atorvastatin for treating NDI in a population of lithium users.

## Methods

### Study design and study sample

This is a double-blind, placebo-controlled RCT examining the effectiveness of atorvastatin versus placebo in the treatment of lithium-induced NDI. The study will take place at three tertiary care mental health sites in Montreal, Canada: Douglas Mental Health University Institute (DMHUI), Jewish General Hospital (JGH) and McGill University Health Centre (MUHC).

The study will involve treatment with atorvastatin (20 mg/day) or placebo over a period of 12 weeks. We are aiming to recruit sixty individuals between the ages of 18 and 85 with any psychiatric diagnosis who are currently taking lithium for at least 2 months and have a urine osmolality < 600 mOsm/kg after 10-h water restriction. We opted for a urinary osmolality of < 600 mOsm/Kg: patients with < 600 mOsm/kg are at risk of future NDI-related complications, such as CKD and they constitute a large percentage of lithium users (50% of patients). Other studies have used similar, if not less conservative definitions for NDI [25], which are nonetheless of clinical importance and utility [15]. Other authors have used similar or more liberal definitions in NDI treatment studies [25].

The full inclusion and exclusion criteria can be found in Table 1.

**Table 1:** Inclusion and exclusion criteria

Inclusion Criteria	Exclusion Criteria
Between ages 18–85	Already taking statins (at least 6 weeks prior to screening)
Bipolar Diagnosis in any phase of illness: euthymic, depressed or hypomanic. Any schizoaffective diagnosis.	History of adverse reaction to statins; allergy to statins
Diagnosed with NDI, defined by routine 10 h water restriction urine osmolality < 600 mOsm/Kg	Contraindications to statin use: pregnancy or lactation, use of fibrates and heavy alcohol use
Currently taking lithium medication (at least 2 months)	Inability to give consent or deemed to have moderate-severe cognitive disturbances by treating clinician
Able and willing to give informed consent	Active liver disease
	Baseline LDL level < 1.5

### Recruitment procedures

**Fig. 1:**
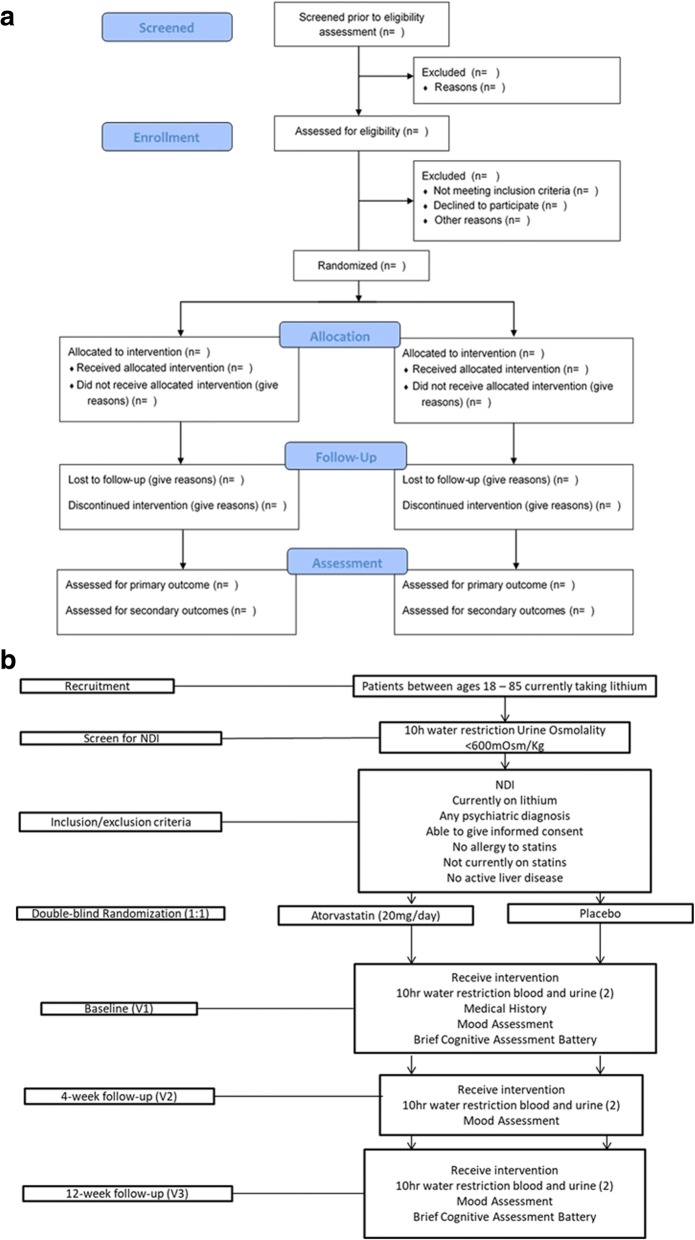
**a** Flow chart – Flow of Study Participants through the RCT. **b** Consort diagram for reporting randomized controlled trials (Eldridge et al., 2016 [40])

This study has been approved by the local institutional review board (IRBs) of each participating center. Participants are introduced to the study by their treating physician. Interested participants meet with the study research assistant for more details and written informed consent. Patients are then screened for NDI (< 600 mOsm/Kg after 10 h of water-restriction) by their treating clinicians as part of routine clinic visits or by the research assistant in a subsequent visit. Participant flow through the study is described in Fig. 1a and b.

### Overview of study procedures

In this double-blind RCT, sixty patients will receive 12 weeks of either treatment (20 mg/day atorvastatin) or placebo. The study will require three visits at baseline, 4-week and 12-week follow-up, for which participants will be compensated. During each visit, blood and urine samples after 10 h of water restriction and 12 h of fasting will be requested. In addition to the primary and secondary outcomes, patients will be monitored for safety using blood tests for liver function (AST and ALT), cholesterol (e.g. low-density lipoprotein (LDL)) and lithium toxicity (lithium levels, estimated glomerular filtration rate (eGFR)). Patients will also complete questionnaires about their medical history and mental health. Mood assessments for depression and mania symptoms will be completed at baseline, 4-week and 12-week follow-up, including the Montgomery-Asberg Depression Rating Scale (MADRS) [26], Young Mania Rating Scale (YMRS) [27], and the Quick Inventory of Depressive Symptoms, Self-report-16 (QIDS-16) [28], Altman Self-Rating Mania Scale (ASMRS) [29].

The primary outcome is change in 10 h water restriction urine osmolality (mOsm/kg) and will be assessed at baseline, 4-week and 12-week follow-up visits (Table 2).

**Table 2:** Baseline and follow-up visits schedule of events and assessments

Assessment	Screen	Baseline	4-week	12-week
Blood	√	√	√	√
Urine osmolality	√	√	√	√
Medical History	√	√		√
Mood Assessment		√	√	√
1.MADRS		√	√	√
2.YMRS		√	√	√
3. QIDS		√	√	√
4. Altman self-rating mania scale		√	√	√

### Randomization and concealment

After eligibility is confirmed and patients have given consent, patients will be randomized in a 1:1 ratio to receive either 20 mg per day of atorvastatin (treatment group) or placebo (control group). We are using a stratified randomization, with two strata: 1. Diagnosis (bipolar disorder, other diagnosis (e.g. depression, schizoaffective disorder), and 2. McGill hospital site (Douglas Mental Health Institute, Jewish General Hospital (JGH), and McGill University Health Centre (MUHC)). An independent entity, the Lady Davis Institute Data Management Unit is responsible for setting up data management software (Redcap) and randomization. Allocation of participants to groups is concealed in an envelope that only the pharmacy at the Douglas Institute has access to. The patients, clinicians, researcher and outcome assessors will all be blind to the treatment allocation. An independent statistician will perform the statistical analyses.

### Assessments

#### Outcomes measures

Our primary outcome will be urinary osmolality after 10-h water restriction. This short-period water restriction was selected given that longer periods of water restriction are often poorly tolerated by patients, especially those on lithium treatment [30]. Clinically, the 10-h water restriction assessment of urine osmolality is often used and is highly specific and sensitive for NDI [25, 31, 32]. We will assess the raw urine osmolality value at 12-weeks, adjusted for baseline. An increase in urine osmolality (mOsm/Kg) values adjusted for baseline will be indicative of improvements in the kidney’s ability to concentrate urine [33, 34]. We hypothesize that higher osmolality values will be observed compared to baseline in patients randomized to atorvastatin.

We chose measures of aquaporin 2 excretion and self-reports of daily fluid intake as the secondary outcomes. Aquaporins are channels involved in water reabsorption in the collecting ducts of the kidney [35]. Assessment of aquaporin excretion in urine will be indicative of the expression of the aquaporin channels and water reabsorption capabilities of the kidneys, allowing further confirmation of whether: 1) NDI pathology is present and 2) whether it is being modified by atorvastatin treatment. Furthermore, changes in daily fluid intake have been highly correlated with both urinary osmolality and the gold-stand as a characteristic of NDI [30, 31, 36]. Additionally, we supplement these secondary measures with self-reported frequency of nighttime and daytime polyuria.

#### Safety and tolerability measures

Safety and tolerability outcome measures will be assessed including blood tests at routine and follow-up visits to ensure patient health (monitoring atorvastatin-related adverse effects). Blood tests will allow the monitoring of serum lithium levels, lipid profile (including LDL levels), creatinine kinase, liver function enzymes, calcium, thyroid stimulating hormone, and electrolytes. Patient reported symptoms – e.g. for myalgias – are also reported. In addition, mood assessments - YMRS, MADRS, QIDS, and AMSRS assessments during follow-up visits will be used to monitor mood changes in patients. YMRS is an eleven-item scale to assess manic symptoms in individuals [27]. MADRS is a ten-item scale for a measure of depressive symptoms [26]. QIDS is a twenty-item self-report measure on depression [28], whereas the ASRMS is a five-item self-report measure for manic symptoms [29].

#### Baseline demographic and clinical characteristics

We collect information on a number of baseline demographic and clinical characteristics, including: age, sex, body mass index (BMI), psychiatric and other medication history including duration of lithium use and dosage. Of these factors, age, lithium-level, duration, dosage and anti-psychotic use have been associated with NDI [17, 30].

#### Planned analysis

We will use independent two-tailed Student’s t-test (or non-parametric equivalent) to compare the primary outcome (raw score values of urinary osmolality at 12-weeks, adjusted for baseline) and secondary outcomes (self-reported fluid intake and variation in aquaporin 2 protein abundance in urinary exosomes at 12-week follow-up, adjusted for baseline) between the treatment (atorvastatin) and control (placebo) groups. The consensus in the statistics RCT literature is that clinical trial outcomes should not be “change from baseline”, but rather the raw score of an outcome, adjusted for the baseline value [37]. Randomization is expected to balance potential confounders between the treatment and placebo groups since both groups are anticipated to have roughly equal baseline characteristics, which we will assess using t-tests (or non-parametric equivalent) and chi-squared tests, as appropriate. However, in the event that any important baseline characteristic differs between groups, we will use multiple linear regression to control for that particular characteristic.

#### Sample size justification

On repeated measures ANOVA, 60 study completers will allow us to observe an effect size of 0.34 at two-tailed alpha = 0.05 and Power (1-Beta) =0.8 [38]. In our previous cross-sectional findings in chronic lithium users, the effect size of statins on NDI was 0.33 [21], however that was not an intervention study. We acknowledge that there is relatively little data at the current time to justify the sample size. This RCT will provide the necessary and adequate data to plan a subsequent larger RCT [39].

## Discussion

This paper described the methodology of a clinical trial to assess the effectiveness of atorvastatin in treating NDI in lithium users. If atorvastatin is ultimately found to be effective for NDI, some of the risk associated to CKD, hypernatremia and AKI attributable to lithium could be attenuated, allowing a greater number of persons to use lithium more safely. Minimizing these risks of kidney disease associated with lithium use is important because it could potentially prevent acute and chronic medical health care services related to renal disease and psychiatric illness, possibly saving significant personal and health care costs worldwide.

The present study is the first RCT of atorvastatin in the treatment of Li-induced NDI. This study represents an advance in methodological rigor compared to previous studies examining the treatment of NDI [17]. These studies were similar in design to the present study, however these used very small sample sizes (*n* = 9, *n* = 11) [18, 25]. Hydrochlorothiazide (HCTZ), other diuretics, non-steroidal anti-inflammatory drugs (NSAIDs), and aspirin (ASA) have also been tried in NDI, but the studies supporting their use are almost exclusively from small uncontrolled trials/chart reviews [17]. In addition, there are limitations to the use of amiloride, diuretics, NSAIDs and other medications previously tested to treat NDI: these medications can lead to elevations in lithium levels contributing to increased risk of lithium-associated CNS and acute renal toxicity. We present an RCT with comparatively large sample size (*n* = 60), which will provide preliminary data to guide larger-scale international collaborative study.

### Limitations

There are some limitations to this proposed study. For our primary outcome, urine osmolality, we used a threshold of < 600 mOsm/Kg after 10 h of water restriction to define NDI and allow patients to enter the study. NDI is ideally more conservatively measured with an even lower urine osmolality threshold that is more specific/sensitive for NDI. However, a urine osmolality < 600 mOsm/Kg is a clinically prevalent entity (50% of patients), associated with poor renal outcomes and is nonetheless of clinical importance and utility [15]. Other trials have used similar thresholds (e.g. < 700 mOsm/kg) under similar 8–10-h water restriction periods, has been termed as “partial NDI” [25]. The overnight water restriction urine osmolality has relatively high sensitivity/specificity for NDI compared to the gold-stander 24-h urine collection, particularly in combination with self-reported fluid intake, which we are also measuring [31]. In addition, we plan to complete a sub-group analysis of patients with a urine osmolality < 300 mOsm/Kg.

## Conclusion

To our knowledge, this is the first planned RCT for the treatment of NDI using atorvastatin. Future trials could 1) further assess the effectiveness of atorvastatin in treating lithium-induced NDI in a larger sample and 2) examine atorvastatin in the prevention of lithium-induced NDI and CKD. If found effective, atorvastatin could ultimately be combined with lithium regimens to treat/prevent NDI, which in turn could reduce the risk of CKD and other renal adverse events in lithium users. This could potentially allow clinicians to prescribe lithium to a larger number of patients with bipolar disorder, depression and other psychiatric/neurological disorders: Both maximizing positive potential therapeutic benefits of lithium, while minimizing renal adverse events.

## Abbreviations

AKI: Acute Kidney Injury
ALT: Alanine Aminotransferase Test
ANOVA: Analysis of Variance
AQP2: Aquaporin 2
ASA: Aspirin
ASRMS: Altman Self-Rating Mania Scale
AST: Aspartate Aminotransferase Test
BMI: Body Mass Index
CKD: Chronic Kidney Disease
eGFR: Estimated Glumerular Filtration Rate
HCTZ: Hydrochlorothiazide
IRB: Institutional Review Board
LDL: Low Density Lipoprotein
MADRS: Montgomery Asberg Depression Rating Scale
NDI: Nephrogenic Diabetes Insipidus
NSAIDs: Non-Steriodal Anti-Inflammatory Drugs
QIDS: Quick Inventory of Depressive Symptoms
RCT: Randomized Controlled Trial
SCIP: Screen for Cognitive Impairment in Psychiatry
UOsm: Urinary Osmolality
YMRS: Young Mania Rating Scale
